# Disparities in Care for Low-Income Patients with Cirrhosis: Implementing an Innovative Outpatient Clinic for Refractory Ascites in a Safety Net Hospital

**DOI:** 10.1007/s11606-024-08675-0

**Published:** 2024-02-20

**Authors:** Shadi Dowlatshahi, Jennifer Koh, Annasha Vyas, Wendy J. Mack, Barbara J. Turner

**Affiliations:** 1 Department of Hospital Medicine, Los Angeles General Medical Center, Los Angeles, CA USA; 2 Department of Medicine, Keck Medical Center of University of Southern California, Los Angeles, CA USA; 3 Department of Population and Public Health Sciences, Keck School of Medicine, USC, Los Angeles, CA USA; 4 Gehr Family Center for Health Systems Science and Innovation, Keck School of Medicine of USC, Clinical Sciences Center, Los Angeles, CA USA

**Keywords:** cirrhosis, health care disparities, safety net hospitals, paracentesis, ambulatory care facility, Hispanic Latino, quality improvement science

## Abstract

**Background:**

Disparities in life-saving interventions for low-income patients with cirrhosis necessitate innovative models of care.

**Aim:**

To implement a novel generalist-led FLuid ASPiration (FLASP) clinic to reduce emergency department (ED) care for refractory ascites.

**Setting:**

A large safety net hospital in Los Angeles.

**Participants:**

MediCal patients with paracentesis in the ED from 6/1/2020 to 1/31/2021 or in FLASP clinic or the ED from 3/1/2021 to 4/30/2022.

**Program Description:**

According to RE-AIM, adoption obtained administrative endorsement and oriented ED staff. Reach engaged ED staff and eligible patients with timely access to FLASP. Implementation trained FLASP clinicians in safer, guideline-based paracentesis, facilitated timely access, and offered patient education and support.

**Program Evaluation:**

After FLASP clinic opened, significantly fewer ED visits were made by patients discharged after paracentesis [rate ratio (RR) of 0.33 (95% CI 0.28, 0.40, *p* < 0.0001)] but not if subsequently hospitalized (RR = 0.88, 95% CI 0.70, 1.11). Among 2685 paracenteses in 225 FLASP patients, complications were infrequent: 39 (1.5%) spontaneous bacterial peritonitis, 265 (9.9%) acute kidney injury, and 2 (< 0.001%) hypotension. FLASP patients rated satisfaction highly on a Likert-type question.

**Discussion:**

Patients with refractory ascites in large safety net hospitals may benefit from an outpatient procedure clinic instead of ED care.

## INTRODUCTION

The prevalence of cirrhosis in the USA has risen 1.5- to twofold in the past two decades and, despite advances in care, age-adjusted mortality has continued to rise^1^ resulting in over 56,000 deaths annually.^2^ The prevalence of cirrhosis has been reported to be higher in non-Hispanic blacks and Mexican Americans, low-income persons, or those with less than a 12th grade education.^3^ These vulnerable populations experience disparities in life-saving interventions for cirrhosis such as the transjugular intrahepatic portosystemic shunt (TIPS)^4^ procedure for portal hypertension and liver transplantation.^5–7^ Without these interventions, patients with cirrhosis often develop ascites that is refractory to medical interventions and requires repeated paracentesis.

The need for paracentesis places significant demands on emergency departments (ED) and hospitals.^8,9^ Reducing this urgent care utilization has been proposed as a quality-of-care measure.^10^ Alternative outpatient models of care have emerged for managing ascites such as paracenteses performed by interventional radiologists (IR).^11–13^ Yet safety net institutions, defined by the Institute of Medicine as primarily serving patients with no insurance or Medicaid, may lack outpatient IR services due to staffing, space, and/or financial barriers.^14^ For veterans, the alternative of an outpatient procedure clinic staffed by hospitalists has been described.^15^ To our knowledge, this model has yet to be adopted in safety net hospitals despite their having one-third of all hospitalizations for cirrhosis and its complications in the USA.^16^ This quality improvement science project describes logistics and outcomes of implementing an innovative generalist-led outpatient clinic for paracentesis in a large safety net hospital during the COVID-19 pandemic with the objectives of relieving the burden on the ED and safely serving predominantly Latino patients with cirrhosis.

### Setting and Participants

A dedicated outpatient FLuid ASPiration (FLASP) clinic was launched in March 2021 by one hospitalist in a large safety net hospital serving the low-income population of Los Angeles county. Eligible patients for FLASP clinic had received at least one paracentesis in the health system’s ED or inpatient setting in the previous month and needed regular paracentesis for ascites. Patients needed to be insured by MediCal and served by the LA Department of Health Services network. In accordance with American Association for the Study of Liver Diseases (AASLD) guidelines,^17^ FLASP clinic treated patients with thrombocytopenia, prothrombin time prolongation, and/or anticoagulation or antiplatelet therapy.

### Program Description and Analysis

According to the RE-AIM framework,^18^ adoption of the clinic was conceptualized by the hospitalist to address the gap in care for patients repeatedly receiving paracentesis in the ED, despite long waits during the COVID-19 pandemic. The chief medical officer (CMO) concurred and endorsed establishing the clinic but approval was predicated on the clinic reducing demand for paracentesis in the ED.

FLASP clinic was initially staffed by a hospitalist director and a dedicated nurse. After the clinic opened, data on reduction in ED visits for paracentesis prompted the CMO to support additional staffing by multiple generalist (staff) physicians and, starting July 1, 2021, an intern rotating in clinic. A NP joined the team on September 1, 2021. To meet increasing demand for paracentesis services, weekday clinic hours rose from half-day (8am to 12 pm) to full day (8am to 4 pm). Physicians staffed the clinic half-day and the NP staffed full day.

To promote reaching eligible patients, the FLASP clinic director delivered a brief in-person or video orientation to ED clinicians and staff with a handout about patient eligibility, FLASP procedures, and referral logistics. Non-ED staff physicians and residents received this information via email. To facilitate timely access, ED staff used the electronic medical record (EMR) to schedule a FLASP clinic appointment within 1 or 2 days. Bilingual clerks and nursing staff assisted patients with FLASP appointment logistics and reminders. The FLASP staff also reviewed monthly reports from the EMR system of paracenteses in the ED and called potentially eligible patients to offer care in the clinic. Once the clinic was more established, physicians in internal medicine and specialty clinics (i.e., hepatology and gynecology/oncology) also referred patients to the clinic.

Implementation focused on performing paracentesis safely through rigorous training and observation following AASLD guidelines.^17,19^ The clinic director delivered a 1-week training program for clinic physicians, interns, and NP as well as watched each clinician perform at least five paracenteses. The protocol for paracentesis required using ultrasound guidance (details available upon request). To limit albumin replacement and acute kidney injury (AKI), 5 L or less ascitic fluid was removed per AASLD guidelines.^17^ Vital signs were monitored and laboratory tests evaluated after the procedure.

To reduce the frequency of paracenteses, staff offered information in Spanish or English to patients about dietary requirements and support for medication adherence. To promote consideration of patients’ receiving the TIPS procedure, longitudinal care by FLASP clinic facilitated shared management with hepatology.

To evaluate the effectiveness of FLASP clinic on reducing ED utilization for paracenteses and patient engagement, monthly ED rates were modeled using univariate Poisson regression, with the monthly paracentesis count as a dependent variable and total monthly ED visits as a model offset variable. Rates with 95% confidence intervals (CI) were reported as numbers of paracenteses per 1000 ED visits. To test for changes in ED paracentesis rates following FLASP clinic implementation, we created a binary variable for each pre- and post-clinic study month with results expressed as ED paracentesis rate ratios (95% CI) comparing pre- to post-implementation months. Median and interquartile range of responses to an anonymous patient Likert-type question about satisfaction were also calculated. This quality improvement science project was approved by the University of Southern California Institutional Review Board (UP-20–01435).

### Program Evaluation

A key FLASP clinic goal was reducing demand for paracentesis in the ED. From 6/1/2020 to 1/31/2021 before the clinic opened, EMR data identified 172 unique patients who received 416 paracenteses in the ED. Excluding a transitional month, 225 unique patients were identified from the EMR as receiving paracentesis in FLASP clinic from 3/1/2021 through 4/30/2022. The patients treated in the ED did not differ significantly from those in the FLASP clinic in demographic or clinical characteristics (Table 1). In both settings, patients’ average age was mid-50 s and less than one-quarter were women. Hispanic/Latinos comprised over 90% of patients. The most common etiology of cirrhosis was alcohol consumption, and the median MELD score was 18 for both groups.

**Table 1:** Patients Receiving Paracentesis Before FLASP Clinic in the Emergency Department (ED) Versus FLASP Clinic Patients

Patient characteristics (*N*)	ED patients (6/2019–2/2021), (172)	FLASP patients (3/2021–4/2022), (225)	*P*-value*
	Percent (*N*)	Percent (*N*)	
Age, years (mean, SD)	55.7 (10.8)	55.5 (10.0)	0.83
Men	119 (69.2)	154 (68.4)	0.87
Hispanic/Latino	154 (89.5)	194 (86.2)	0.32
Cirrhosis etiology			
Alcohol	88 (63.8)	120 (64.5)	0.38
Hepatitis C virus (HCV)	7 (5.1)	15 (8.1)	
Non-alcoholic steatohepatitis (NASH)	7 (5.1)	15 (8.1)	
Mixed (i.e., NASH/alcohol, alcohol/HCV, HCV/NASH)	20 (14.5)	17 (9.1)	
Other (i.e., autoimmune, cryptogenic)	16 (11.6)	19 (10.2)	
Cardiac	9 (5.2)	5 (2.2)	0.11
Malignancy	25 (14.5)	35 (15.6)	0.78
MELD score, median (IQR)^**†**^	18 (10)	18 (10)	0.68

^*^P-value for group comparisons from the chi-square test for categorical variables, *t*-test for age, and Wilcoxon rank sum test for Model for End-Stage Liver Disease (MELD)

^†^MELD assessed in 142 patients in the ED group and in 185 patients in the FLASP group due to lacking INR to calculate MELD

**Figure 1:**
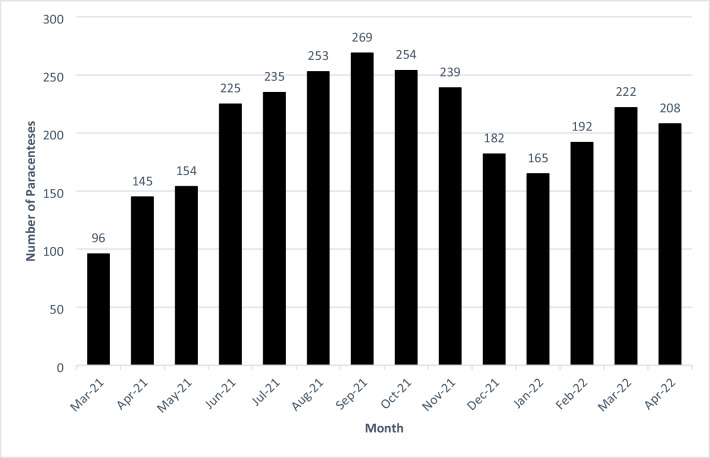
Paracenteses performed per month in the FLASP clinic after opening March 1, 2021 through April 2022.

Utilization of the FLASP clinic grew quickly with monthly paracenteses increasing from 96 in March 2021 to a high of 269 in September 2021 (Fig. 1, Appendix). Mean monthly procedures then stabilized at 203 as patients were increasingly shared with hepatology for consideration of TIPS or liver transplant.

**Figure 2:**
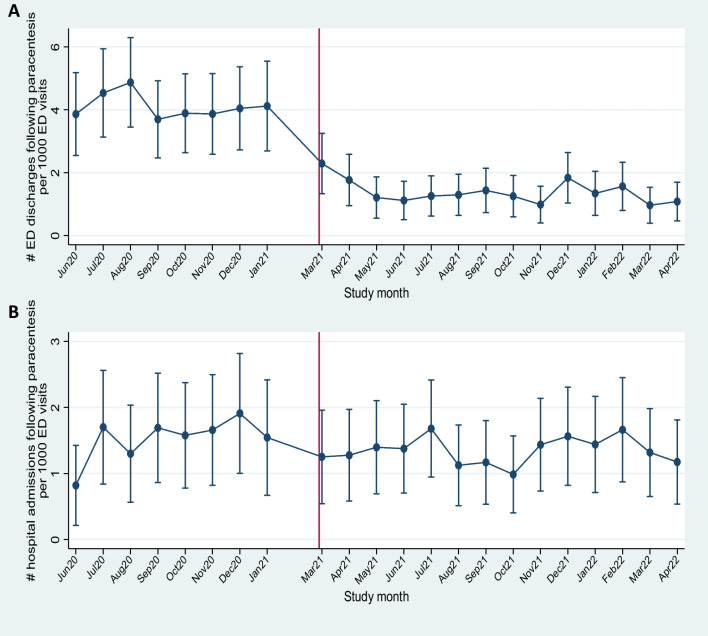
**A** Number of ED visits for paracenteses and discharged per 1000 ED visits (95% CI) discharged, before (June 2020–January 2021) and after the FLASP clinic (Mar 2021–April 2022). The red line indicates when the FLASP clinic was established on March 1, 2021. **B** Number of ED visits for paracenteses and admitted to the hospital per 1000 ED visits (95% CI).

Mean monthly visits for paracentesis per 1000 ED visits declined significantly from 4.11 before the FLASP clinic to 1.37 afterward (*p* < 0.0001) (Table 2). Lower ED utilization was observed for patients discharged after paracentesis (Fig. 2A, Appendix) with a rate ratio of 0.33, 95% CI 0.28, 0.40, *p* < 0.0001 for the comparison of before and after FLASP implementation. Mean monthly ED visits for patients who were hospitalized after the procedure declined from 1.53 per 1000 ED visits before FLASP clinic to 1.35 per 1000 ED visits afterward (Fig. 2B, Appendix) but this change was not significant (rate ratio 0.88, 95% CI 0.70, 1.22; *p* = 0.28) (Table 2).

**Table 2:** Monthly Visit Rate for Paracentesis in the Emergency Department (ED) Before and After Implementation of the FLASP Clinic

Timeframe	Paracenteses in ED followed by discharge Rate (95% CI)*	Paracenteses in ED followed by hospitalization Rate (95% CI)*
Pre-FLASP clinic (per 1000 ED visits)	4.11 (3.64, 4.58)	1.53 (1.24, 1.82)
Post-FLASP clinic (per 1000 ED Visits)	1.37 (1.19, 1.56)†	1.35 (1.16, 1.53)
Rate ratio	0.33 (0.28, 0.40)†	0.88 (0.7, 1.11)

^*^Monthly ED rates modeled using Poisson regression, with the monthly paracenteses count as the dependent variable and total monthly ED visits as a model offset variable. For each study month, a binary variable for pre- vs. post-clinic implementation was created to statistically test for changes in ED paracenteses rates following clinic implementation. Results expressed as ED paracentesis rate ratios (post- compared to pre-implementation), with 95% confidence intervals

^†^*P* < 0.0001

Safety was essential for the FLASP clinic, especially because it trained interns. Over 14 months after launch, 225 patients received 2685 paracenteses in the clinic. Within 1 week after each procedure, analyses of the EMR revealed that 39 (1.5%) were complicated by SBP and 265 (9.9%) by acute kidney injury (AKI) per KDIGO guidelines.^20^ Two cases of hypotension were reported with one (0.0007%) requiring hospitalization. No other peri- or post-procedural complications were identified including abdominal wall hematoma, hemorrhage, organ perforation, and ascitic leakage.

All 40 patients served in FLASP clinic within a 2-week period anonymously responded to a Likert-type question about satisfaction with response options from 1 (very dissatisfied) to 5 (very satisfied). The median rating was 5, with 25th and 75th quartiles both 5. In addition, patient adherence to scheduled visits was high with a “no-show” rate of only 4% to the 2685 FLASP appointments over 14 months.

## DISCUSSION

Safety net hospitals fill a vital role by serving low-income persons.^14,21^ However, persons with cirrhosis at these institutions experience barriers to life-prolonging interventions such as the TIPS procedure and liver transplantation.^22^ Lacking these interventions, refractory ascites requires repeated paracentesis that burdens not only urgent care settings but also the patients themselves.^23^ To improve quality of care for refractory ascites in a large safety net hospital, this study describes implementation of the outpatient FLASP clinic for paracentesis using the RE-AIM framework.^18^ The FLASP clinic performed approximately 2700 paracenteses over 14 months in patients with similar demographic characteristics to those with paracenteses in the ED. The significant two-thirds reduction in the rate ratio of paracenteses in the ED before and after FLASP clinic supports achievement of our effectiveness goal of reducing demand on the ED. Notably, this was accomplished during the COVID-19 pandemic when our Latino urban population had significant need for urgent care.^24^

Regarding lessons learned, implementation of FLASP clinic required first obtaining endorsement from the institution’s leadership and mutually agreeing on the goal of reducing paracenteses in the ED. Another lesson involved using multiple approaches to reach and engage ED staff and eligible patients. The FLASP clinic director personally offered informational sessions and handouts to ED physicians and staff. Housestaff and relevant physicians received emailed announcements about FLASP clinic. EMR procedures were developed for ED staff to make timely FLASP appointments. In addition, FLASP clinic staff contacted patients about appointments and offered education about dietary changes and medication adherence to reduce the frequency of paracentesis. Longitudinal care delivered by clinic staff established shared care with hepatology services for consideration of life-saving interventions.

The FLASP clinic’s team-based care featured attending physicians, a NP, and interns as in Veterans Administration outpatient procedure clinics.^15^ Another lesson from our implementation project was the value of rigorous, protocol-guided training in ultrasound-guided paracentesis and direct observation of clinicians performing the procedure. AKI occurred after only 10% of paracenteses in FLASP clinic compared with AKI rates of 24%^25^ and 66% in a study of paracenteses in outpatient clinics.^15^ To reduce AKI risk even further, the FLASP team recognized that they may have been performing parentheses too frequently and changed to approximately every 2 weeks along with more volume replacement. SBP was observed in 1.5% of FLASP paracenteses versus 2% incidence of SBP in a recent systematic review of outpatient paracenteses.^26^ The FLASP clinic also aimed to improve patient care experience. We found that satisfaction was high on a survey of 2-week sample of consecutive patients.

We acknowledge study imitations. After FLASP clinic opened, ED visits for paracentesis did not decrease significantly for patients who were subsequently hospitalized. This demand persisted in part because Latinos with cirrhosis, in particular, experience complications requiring inpatient care.^27–29^ Similarly, an outpatient IR clinic for paracenteses reported that it reduced ED utilization only for discharged patients but not for those who were directly hospitalized from the ED.^11^ This finding reinforces the need to establish triage protocols to assess patient stability.^10^

FLASP clinic currently performs 60–80 paracenteses weekly in our large hospital. Smaller safety net hospitals may not benefit from a dedicated procedure clinic. According to the Healthcare Cost and Utilization Project (HCUP), safety net institutions are more likely to be medium or large than other non-rehabilitation hospitals.^14^ However, definitions of safety net institutions vary.^21^ In a study of differing qualifications for safety net institutions, the number of large (> 300 bed) hospitals ranged from 223 to 795.^21^ FLASP clinic is most likely generalizable to these institutions. But FLASP clinic has added other procedures such as thoracentesis, joint injections/aspirations, and lumbar puncture that could increase the value of such a clinic for smaller institutions.

We do not have information about paracenteses performed in other EDs after the FLASP clinic opened and may have missed complications occurring weeks after the procedure or treated in other settings. Yet few such urgent care settings exist for our low-income patients. In addition, without outpatient IR paracenteses in our institution, we cannot compare outcomes to those of FLASP clinic. An inpatient study comparing bedside ultrasound-guided paracentesis to IR paracentesis reported better outcomes with the former.^30^ Overall, the FLASP clinic for low-income patients with chronic ascites delivered safe, well-accepted care that has equivalent if not better outcomes compared with reports from other outpatient models of care.

In conclusion, the FLASP clinic successfully reached similar patients to those seeking ED care and met the leadership’s expectations of reducing ED paracenteses. Relatively few complications occurred in FLASP patients and patient satisfaction was high. Thus, FLASP clinic offers a valuable outpatient model for larger safety net institutions to address needs of low-income patients with cirrhosis and the serious complication of refractory ascites.^31^

## Data Availability

Deidentified data are available upon request from Dr. Mack.

## Appendix

Figs. 1 and 2
